# Role of zonisamide in advanced Parkinson’s disease: a randomized placebo-controlled study

**DOI:** 10.1007/s10072-024-07396-w

**Published:** 2024-02-20

**Authors:** Mohamed Essam, Eman Hamid, Eman Abushady, Mahmoud El-Balkimy, Angelo Antonini, Ali Shalash

**Affiliations:** 1 Department of Neurology, Faculty of Medicine, Ain Shams University, 38 Abbassia Square, Cairo, Egypt; 2 Parkinson and Movement Disorders Unit, Study Center for Neurodegeneration (CESNE), Department of Neuroscience, University of Padua, 35131 Padua, Italy

**Keywords:** Zonisamide, Parkinson̕ s disease, Wearing off, Nonmotor symptoms, Quality of life

## Abstract

**Background:**

Zonisamide (ZNS) has shown some efficacy in motor symptoms of PD; however, more evidence is lacking, and its effects on nonmotor symptoms (NMSs) and quality of life (QoL) remain to be investigated. This randomized double-blinded placebo-controlled crossover study investigated the effect of ZNS on motor and NMS symptoms and QoL in advanced PD.

**Methods:**

PD patients with Hoehn and Yahr stage ≥ 2 (“On” state) and at least 2 h off time daily were randomized to groups: ZNS 25 mg, ZNS 50 mg and placebo. Groups were assessed at baseline and at the 1- and 3-month follow-ups. The primary endpoint was the change in the total MDS-UPDRS III “On”, while the secondary endpoint was the change in the total and parts I and IV MDS-UPDRS, Nonmotor Symptoms Scale and Parkinson’s disease questionnaire-39 at the final assessment.

**Results:**

Sixty-nine patients were assessed for efficacy at the 1-month follow-up, and 58 patients were assessed at the 3-month follow-up. The primary endpoint showed significant improvement in the ZNS 25 mg group compared to the placebo group (*p* = 0.009). At the final assessment, the ZNS 25 mg group showed significant improvement of total and part VI MDS-UPDRS, bradykinesia, tremor and functional impact of fluctuations compared to placebo. There was no change in dyskinesia, NMSs, QoL or side effects except for sedation.

**Conclusion:**

ZNS has a favourable effect on motor symptoms in patients with wearing off as adjunctive therapy with other dopaminergic drugs, with no exacerbation of dyskinesia and a limited impact on NMSs and QoL.

**Trial registration:**

Clinicaltrials.gov, NCT04182399, in 24/11/2019.

**Supplementary Information:**

The online version contains supplementary material available at 10.1007/s10072-024-07396-w.

## Introduction

Parkinson’s disease (PD) has been identified as the fastest-growing neurodegenerative disorder worldwide, with a rising global burden [1]. Disease burden increases in advanced stages with the development of long-term levodopa motor complications, wearing off and levodopa-induced dyskinesia. Although different options exist, improving wearing off without increasing dyskinesia is still an unmet need for patients with advanced PD [2]. Additionally, the availability of different therapies for these long-term complications is lacking among several countries, particularly African countries, implying the need for affordable and available therapies [3].

Zonisamide (ZNS) (1,2-benzisoxazole-3-methanesulfonamide) is an antiepileptic drug that was approved in Japan in 1989 to treat partial and generalized seizures and received FDA approval in 1998 to treat partial epilepsy in adults [4]. Accidentally, ZNS improved symptoms of PD in a Japanese patient with PD who developed a seizure disorder [5]. ZNS has multiple functions, including inhibition of Na channels, T-type calcium channels, monoamine oxidase-B (MAO-B) activity and striatal-δ1-receptor associated gamma-aminobutyric acidergic transmission, activation of dopamine synthesis and dopamine release [6, 7]. Both the dopaminergic and nondopaminergic functions of ZNS may contribute to its effects on PD; however, the exact mechanism of action is still not fully understood. ZNS has a long half-life (25 mg, half-life 90 h); therefore, it was expected to improve the wearing-off phenomenon. ZNS is available in several countries, including Egypt, and is licenced as an adjunctive antiepileptic for partial seizures.

Three double-blinded placebo-controlled studies (phase 2, 2b/3 and 3 studies) were conducted in Japan and showed that ZNS 25 mg was safe and efficacious in improving motor symptoms of PD with a low incidence of adverse effects [8, 9]. In another Japanese randomized double-blinded placebo-controlled study, ZNS 50 mg/day was efficacious in reducing “Off” time (0.719 ± 0.179 h compared with 0.011 ± 0.173 h for placebo, *p* = 0.005) in PD patients wearing off without increasing troublesome dyskinesia [7].

However, evidence of its effect on nonmotor symptoms (NMSs) is lacking [10]. Recently, a study showed a beneficial effect of ZNS on sleep parameters in people with PD using polysomnography; however, further studies are warranted to confirm its impact on NMS and dyskinesia [11]. Moreover, a genome-wide association study showed the association of ZNS favourable response on wearing off with SNP rs16854023 carriers [12]. Furthermore, most of the studies were conducted among the Japanese population, and similar findings from other clinical trials among other populations are still missing [13].

Therefore, in the current study, we investigated the efficacy of ZNS on motor severity and dyskinesia among Egyptian patients with advanced PD. Moreover, we exploratorily evaluated its effect on NMSs, including cognition and mood.

## Materials and methods

In this randomized double-blinded placebo-controlled study, patients were recruited from the movement disorders clinic at Ain Shams University over a period of 2 years between April 2020 and April 2022. Inclusion criteria were patients older than 18 years diagnosed with PD according to UK Brain Bank diagnostic criteria with motor complications (modified Hoehn and Yahr (H&Y) stage ≥ 2 in “On” state) and at least 2 h “Off” time daily. Exclusion criteria were patients with atypical or secondary parkinsonism, patients who could not perform the tests, patients who were or might be pregnant (childbearing potential females who did not practice effective contraception) and breastfeeding females. Patients were informed of the objectives, procedures and possible benefits and risks of the study and provided written voluntary consent, which also allowed patients to withdraw from the study at any time without any consequences. The study was approved by the Ethical Review Committee, Ain Shams University, in accordance with the Helsinki Declaration and was registered in clinicaltrials.gov, NCT 04182399.

Eligible patients were randomly assigned to one of three groups: ZNS 25 mg, ZNS 50 mg and placebo group. The drug was provided in capsules with identical shape and was given to patients in numbered boxes to ensure patient blinding. These boxes were prepared by an operator who used a computerized programme to perform the randomization. The assessment was performed by a different operator who was blinded to the patient groups. In the ZNS 50 mg group, ZNS was started at 25 mg once daily for 1 week and then increased to 50 mg once daily to ameliorate side effects. The dosage and regimen of ongoing antiparkinsonian drugs and other drugs that may affect PD symptoms remained unchanged 1 month before and throughout the study period.

Amid the COVID pandemic and dropouts, we continued the study in a crossover design, with a washout period of 1 month before the crossover stage. Patients who gave written voluntary consent to enter the crossover stage were rerandomized again to different study groups. Following patients’ approval and providing written consent, patients from the placebo group were rerandomized to the 25 mg or 50 mg group, while patients from the ZNS 25 or 50 mg group were rerandomized to the placebo group blindly. A generic form of ZNS (Convagran) and a placebo with a similar shape were used and provided by a national company (Mash Premiere Pharmaceutical Company). Both the patient and the physician were blinded to the medication given and the group of each patient.

Recruited patients were subjected to a comprehensive medical history, neurological examination, demographic data and clinical characteristics (age at onset, duration of illness, received medications and levodopa equivalent daily dose (LEDD)). Patients were assessed at baseline and follow-ups at 1 and 3 months after treatment by a blinded rater using the Movement Disorders Society-Unified PD Rating Scale (MDS-UPDRS), H&Y scale and Schwab and England-Activities of Daily Living Scale (S&E-ADL) in “On” and “Off” states [14]. Dyskinesia was assessed with the MDS-Unified Dyskinesia Rating Scale (MDS-UDysRS) [15]. Gait assessment was performed with the new freezing of gait questionnaire (NFOG) [16], 10-m walk test (10-MWT) [17] and Timed Up and Go test (TUG) [18]. Balance was evaluated by the Berg Balance Scale (BBS) [19], NMSs were assessed with the Non-Motor Symptoms Scale (NMSS), and quality of life (QoL) was assessed with the PD questionnaire (PDQ-39) (Arabic version) [20]. The Mini-Mental State Examination (MMSE) [21] was used to assess cognitive functions. Depression and anxiety were assessed by the Arabic version of the Beck Depression Inventory (BDI) [22] and the Hamilton Anxiety Rating Scale (HAM-A) [23], respectively. During follow-up visits, vital signs were assessed and side effects were recorded via a side effect checklist that was prepared according to the most common side effects of ZNS. Patients were allowed to report any unusual side effects during the study period.

The primary endpoint was the change in the total MDS-UPDRSIII “On” from baseline at the 3-month assessment, while the secondary endpoints included the change in the total and parts I and IV MDS-UPDRS, NMSS and PDQ-39 scores at the final assessment. Using an online sample size calculator (https://clincalc.com/stats/samplesize.aspx) with the primary endpoint was continuous (average MDS-UPDRS III), 16 subjects per group will provide a power of 80% with an alpha error rate of 0.05 according to the calculation equation below.

$$\upkappa =\frac{{n}_{2}}{{n}_{1}}=1$$

$${n}_{2}=\frac{(\upsigma \begin{array}{c}2\\ 1\end{array}+\upsigma \begin{array}{c}2\\ 2\end{array}/{\rm K}\left({\text{z}}1-\frac{\mathrm{\alpha }}{2}+{\text{z}}1-\upbeta \right)\begin{array}{c}2\\ \end{array}}{{\Delta }^{2}}$$

$${n}_{1}=\frac{\left({25}^{2}+{25}^{2} /1\right)\left(1.96+1.04\right) \begin{array}{c}2\\ \end{array}}{{25}^{2}}$$

$${n}_{1}=18$$

$${n}_{2}=K* {n}_{1}=18$$

### Statistical analysis

The data were analysed using IBM SPSS software package version 25.0 (Armonk, NY: IBM Corp). According to the distribution of normality using the Shapiro‒Wilk test, quantitative data are described as the mean ± standard deviation or median and interquartile range (IQR). Categorical data are described as frequencies (percentages) and were compared using the chi-square test. The Kruskal‒Wallis test was used to compare independent variables, while the Friedman test was used to compare related variables. A *p* value of 0.05 or less was considered statistically significant, except for the Wilcoxon test following the Friedman test, which was considered significant if < 0.017 due to Bonferroni correction.

## Results

### Patient randomization

**Fig. 1:**
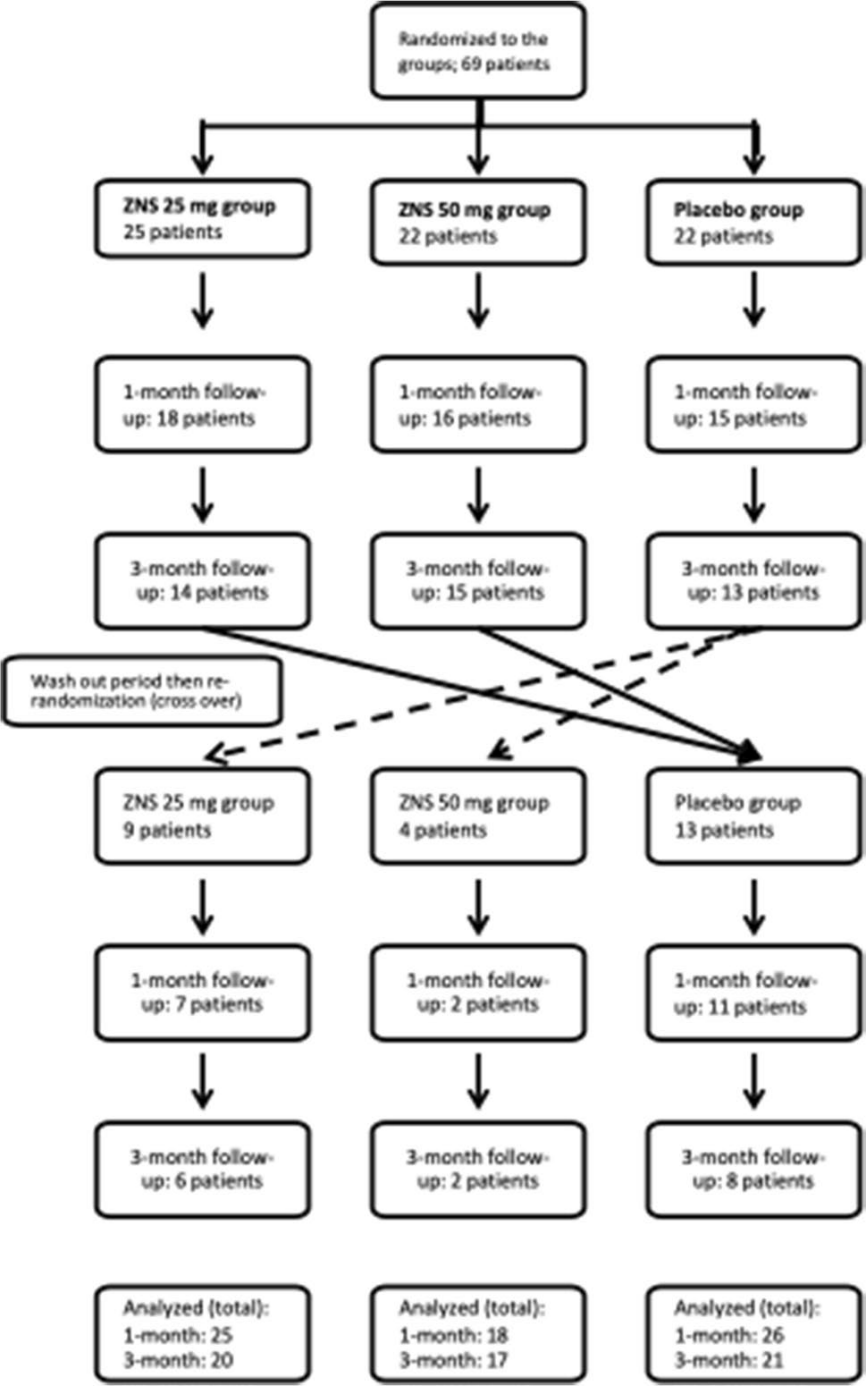
Randomization and distribution of the study population

A total of 69 patients were enrolled in the study and underwent randomization into the 3 groups. Forty-nine patients were assessed at the first follow-up visit (1 month), and 42 patients were assessed at the 2nd follow-up visit (3 months). After a washout period of at least 1 month, 26 patients out of 42 patients who continued until the 3-month follow-up underwent crossover and were rerandomized again to one of the other two groups. Of the 26 crossover patients, 20 patients were assessed at 1 month and 16 patients were assessed at 3 months. Therefore, the total number of patients analysed for efficacy was 69 patients at the 1-month follow-up (25 patients in the ZNS 25 mg group, 18 patients in the ZNS 50 mg group and 26 patients in the placebo group) and 58 patients at the 3-month follow-up (20 patients in the ZNS 25 mg group, 17 patients in the ZNS 50 mg group and 21 patients in the placebo group) (Fig. 1). The most common causes of dropouts were patient withdrawal (17 patients), protocol violation (9 patients), adverse effects and drug intolerance (8 patients) and exacerbation of PD symptoms (3 patients). Adverse effects that led to drug discontinuation were sedation (3 patients in the ZNS 25 mg group, 2 patients in the ZNS 50 mg group and 1 patient in the placebo group), hallucinations (1 patient in the ZNS 25 mg group) and gastric irritation (3 patients (one patient in each group)) (Supplementary Table 1).

### Baseline characteristics

There was no significant difference in the baseline demographics and clinical characteristics of the 3 groups except for sex (*p* = 0.036) and the percentage of patients receiving dopamine agonists in each group (*p* = 0.037). Regarding the MDS-UPRDS, only postural and rest tremors (“On” state) showed significant differences among the 3 groups, with lower scores in the ZNS 50 mg group (*p* = 0.025 and 0.037, respectively) (Table 1, Supplementary Table 2, 3 and 4).

**Table 1:** Baseline demographics and clinical characteristics of the three groups

	ZNS 25 mg (no. = 25)	ZNS 50 mg (no. = 18)	Placebo (no. = 26)	*P* value
Age	55 (13)	50 (12)	53 (11)	0.101
Gender (male/female)	19 (76%)/6 (24%)	9 (50%)/ 9 (50%)	22 (84.6%)/4 (15.4%)	0.036*
AOO	48 (14)	43.5 (13)	47 (12)	0.1
DOI	6 (6)	7 (5)	7 (5)	0.98
LEDD	1050 (868)	1375.5 (516)	1050 (674)	0.27
Dyskinesia at baseline (No (%))	14 (56%)	9 (50%)	17 (65.4%)	0.578
MDS-UPDRS TS OFF	127 (36)	116.5 (63)	120.5 (51)	0.816
MDS-UPDRS TS ON	91 (37)	84.5 (53)	87 (37)	0.535
MDS-UPDRS Part I (nM-EDL)	20 (11)	17 (12)	16.5 (7)	0.654
MDS-UPDRS Part II (M-EDL)	29 (11)	30.5 (10)	27.5 (14)	0.958
MDS-UPDRS Part III OFF	65 (22)	59 (35)	65 (15)	0.397
MDS-UPDRS Part III ON	33 (22)	30 (17)	31 (15)	0.278
MDS-UPDRS Part IV	9 (4)	8 (9)	9 (7)	0.921
Modified H&Y OFF	3 (1)	3 (1)	3 (1)	0.984
Modified H&Y ON	2.5 (0.3)	2.5 (0)	5 (0)	0.876
S&E-ADL OFF	50 (25)	50 (25)	50 (23)	0.834
S&E-ADL ON	80 (25)	80 (23)	80 (10)	0.722
MDS-UDysRS	6 (21)	1.5 (28)	11.5 (22)	0.595
MMSE	27 (5)	27.5 (4)	28.5 (3)	0.451
NMSS	56 (48)	47.5 (30)	55.5 (33)	0.597

Data are shown as median (IQR)) or number (%)

*AOO* Age of onset of illness, *DOI* Duration of illness, *LEDD* Levodopa equivalent daily dose, *MDS-UPDRS* Movement Disorder Society-Unified Parkinson Disease Rating Scale, *TS* Total score, *nM-EDL* Nonmotor aspects of experiences of daily living, *M-EDL* Motor aspects of experiences of daily living, *H&Y* Hoehn and Yahr scale, S*&E-ADL* Schwab and England-Activities of Daily Living, *MDS-UDysRS* Movement Disorder Society-Unified Dyskinesia Rating Scale, *MMSE* Mini-Mental State Examination, *NMSS* Nonmotor Symptoms Scale, *ZNS* zonisamide

^*^P value is significant if < 0.05

### Outcome of ZNS in different groups

At the 1- and 3-month follow-ups, patients in the ZNS 25 mg group showed significant improvements in total MDS-UPDRS “On” (*p* = 0.027) and “Off” scores (*p* = 0.008 and 0.002), motor part of MDS-UPDRS “On “ (*p* = 0.022 and 0.002) and tremor subscore “On “ (*p* = 0.013 and 0.027) compared to baseline scores. Significant improvement in bradykinesia “On” scores was detected at the 3-month follow-up (*p* = 0.003). Analysis of tremor subtypes showed significant improvement of postural tremor in the “Off” (*p* = 0.007 and 0.012 at 1 and 3 months, respectively) and “On” states (*p* = 0.007 and 0.008 at 1 and 3 months, respectively) (Supplementary Table 5). Other parameters did not show significant changes, including dyskinesia scores, NMSs and QoL, compared to baseline scores (Supplementary Table 6).

**Fig. 2:**
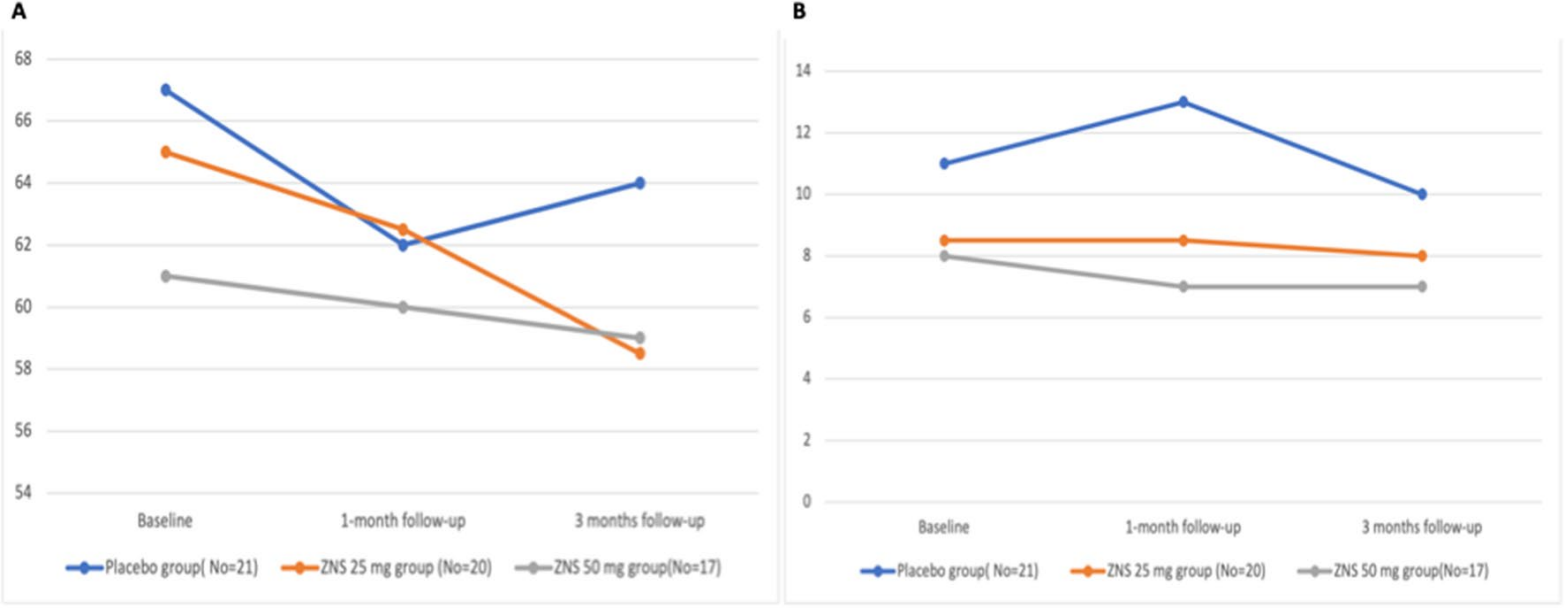
**A** Mean MDS-UPDRS-III scores at different assessments within different groups (significant in the ZNS 50 mg group at 3 months (*p* = 0.003)). **B** Mean MDS-UPDRS-IV scores at different assessments within different groups (significant in the ZNS 50 mg group (*p* = 0.001) at 1 and 3 months)

Among the ZNS 50 mg group, significant improvements in total MDS-UPDRS-Off (*p* = 0011), motor part-Off (*p* = 0.003), rigidity Off (*p* = 0.045) and functional impact of motor fluctuation (*p* = 0.045) were detected at 3 months compared to baseline (Fig. 2A). The MDS-UPDRS part IV showed significant improvement at the 1- and 3-month follow-ups (*p* = 0.005 and 0.012, respectively) (Fig. 2B) (Supplementary Table 7). However, there was a significant worsening of the gastrointestinal tract (*p* = 0.030) and mood/cognition (*p* = 0.039) subscores at the 3-month follow-up. The BDI, HAM-A and PDQ-39 scales did not show significant changes at the 1- and 3-month follow-ups, except for stigma at 3 months (*p* = 0.014) (Supplementary Table 8). The placebo group did not show significant changes from baseline at follow-up, except for total PDQ-39 at 1-month follow-up (*p* = 0.004) (Supplementary Table 9, 10).

### Comparisons of changes between different groups

At 1-month follow-up, there was a significant improvement in total MDS-UPDRS scores “On” among the ZNS 25 mg and ZNS 50 mg groups compared to the placebo group (*p* = 0.007 and 0.021, respectively), significant improvement in tremor “On” scores among the ZNS 25 mg group compared to the placebo group and improvement in total MDS-UPDRS part IV scores among the ZNS 50 mg group compared to the placebo group (*p* = 0.010) (Table 2). Otherwise, there were no significant changes in other scores, including dyskinesia, NMSS, HAM-A, BDI and PDQ-39, from baseline among the 3 groups at the 1-month follow-up (Table 2, Supplementary Table 11).

**Table 2:** Comparison of motor, dyskinesia, gait and balance changes from baseline between the three groups at 1-month follow-up

	ZNS 25 mg (no. = 25)	ZNS 50 mg) (no. = 18)	Placebo (no. = 26)	Kruskal–Wallis H	ZNS 25 mg vs ZNS 50 mg	ZNS 25 mg vs placebo	ZNS 50 mg vs placebo
				*P* value	*P* value	*P* value	*P* value
MDS-UPDRS TS OFF	3 (10)	6 (16)	1.00 (8)	0.158			
MDS-UPDRS TS ON	5 (9)	4 (10)	− 0.50 (5)	0.012*	0.834	0.007*	0.021*
MDS-UPDRS Part I (nM-EDL)	0 (3)	2 (4)	− 1 (2)	0.076			
MDS-UPDRS Part II (M-EDL)	2 (3)	2 (5)	1 (3)	0.320			
MDS-UPDRS Part III OFF	1 (7)	5 (11)	1 (5)	0.751			
MDS-UPDRS Part III ON	2 (7)	0 (6)	1 (4)	0.084			
Rigidity OFF	0 (2)	1 (3)	0 (1)	0.392			
Rigidity ON	1 (2)	0 (3)	0 (2)	0.163			
Bradykinesia OFF	1 (3)	0 (5)	0 (2)	0.953			
Bradykinesia ON	1 (4)	1 (2)	0 (3)	0.603			
Tremors OFF	1 (4)	0 (3)	0 (1)	0.39			
Tremors ON	1 (2)	0 (1)	0 (1)	0.009*	0.021*	0.005*	0.663
MDS-UPDRS Part IV	0 (1)	1 (2)	0 (1)	0.012*	0.009*	0.726	0.010*
MDS-UDysRS	− 1 (3)	0 (1)	0 (2)	0.378			
BBS ON	0 (2)	0 (2)	0 (1)	0.675			
NFOG ON	0 (0)	0 (2)	0 (0)	0.015*	0.01*	0.895	0.011*
10-MWT Comfortable speed ON	0.0022 (0.15)	0.024 (0.06)	0.0005 (0.08)	0.549			
10-MWT Maximum speed ON	0.042 (0.17)	0.04 (0.09)	0.017 (0.09)	0.087			

Data are shown as median (IQR)

*MDS-UPDRS* Movement Disorder Society-Unified Parkinson Disease Rating Scale, *TS* total score, *nM-EDL* Nonmotor aspects of experiences of daily living, *M-EDL* Motor aspects of experiences of daily living, *MDS-UDysRS* Movement Disorder Society-Unified Dyskinesia Rating Scale, *10-MWT*: 10 m walking test, *BBS* Berg Balance Scale, *NFOG* new freezing of gait questionnaire, *ZNS* zonisamide

^*^P value is significant if < 0.05

At 3-month follow-up, there was significant improvement in the primary endpoint (MDS-UPDRS III “On”) compared to placebo (*p* = 0.009). There was also a significant improvement of “On” scores of total MDS-UPDRS (*p* = 010), bradykinesia (*p* = 0.004), tremor (*p* = 0.006), part IV (*p* = 0.013) and functional impact of fluctuations (*p* = 0.007) among ZNS 25 mg compared to placebo. The ZNS 50 mg group showed significant improvement in rigidity “Off” (*p* = 0.018) and painful “Off” state dystonia (*p* = 0.048) at 3 months compared to placebo (Table 3). NMSS, HAM-A, BDI and PDQ-39 scores were not changed significantly at the 3-month follow-up among the 3 groups (Supplementary Table 12).

**Table 3:** Comparison of motor, dyskinesia, gait and balance changes from baseline between the three groups at 3-month follow-up

	ZNS 25 mg (no. = 20)	ZNS 50 mg) (no. = 17)	Placebo (no. = 21)	Kruskal–Wallis H	ZNS 25 mg vs ZNS 50 mg	ZNS 25 mg vs placebo	ZNS 50 mg vs placebo
				*P* value	*P* value	*P* value	*P* value
MDS-UPDRS TS OFF	6 (9)	11 (22)	2.50 (13)	0.147			
MDS-UPDRS TS ON	7 (10)	9 (20)	0 (13)	0.033*	0.915	0.010*	0.066
MDS-UPDRS Part I (nM-EDL)	− 1 (5)	0 (4)	− 1 (4)	0.294			
MDS-UPDRS Part II (M-EDL)	1.5 (4)	3 (6)	0 (5)	0.407			
MDS-UPDRS Part III OFF	2 (10)	5 (11)	2 (9)	0.328			
MDS-UPDRS Part III ON	4 (5)	3 (7)	0 (8)	0.035*	0.320	0.009*	0.185
Rigidity OFF	0 (1)	1 (3)	0 (2)	0.038*	0.189	0.124	0.018*
Rigidity ON	1 (3)	0 (3)	0.50 (3)	0.943			
Bradykinesia OFF	0 (4)	3 (6)	1.50 (5)	0.631			
Bradykinesia ON	2 (2)	1 (3)	0 (2)	0.016*	0.186	0.004*	0.182
Tremors OFF	1 (3)	1 (3)	0 (3)	0.247			
Tremors ON	1 (3)	0 (1)	0 (3)	0.009*	0.013*	0.006*	0.688
MDS-UPDRS Part IV	0 (1)	1 (2)	0 (2)	0.031*	0.066	0.013*	0.331
Functional impact of fluctuations	0 (1)	1 (1)	0 (0)	0.026*	0.243	0.007*	0.119
Complexity of motor fluctuations	0 (1)	0 (1)	0 (0)	0.341			
Painful off-state dystonia-	0 (0)	0 (1)	0 (0)	0.047*	0.017*	0.471	0.048*
MDS-UDysRS	0 (1)	0 (5)	0 (3)	0.782			
BBS ON	1 (2)	0 (1)	0.50(3)	0.370			
NFOG ON	0 (1)	0 (2)	0 (1)	0.078			
10-MWT Maximum speed OFF	0.03 (0.13)	0.02 (0.20)	0.03 (0.07)	0.047*	0.067	0.876	0.013*
10-MWT Maximum speed ON	0.016 (0.16)	0.039(0.13)	0.027 (0.17)	0.289			

Data are shown as median, (IQR**)**

*MDS-UPDRS* Movement Disorder Society-Unified Parkinson Disease Rating Scale, *TS* total score, *nM-EDL* nonmotor aspects of experiences of daily living, *M-EDL* motor aspects of experiences of daily living, *MDS-UDysRS* Movement Disorder Society-Unified Dyskinesia Rating Scale, *10-MWT*: 10 m walking test, *BBS* Berg Balance Scale, *NFOG* new freezing of gait questionnaire, *ZNS* zonisamide

^*^P value is significant if < 0.05

### Safety of zonisamide

Seventeen patients (50%) in the ZNS 25 mg group, 17 patients (65.38%) in the ZNS 50 mg group and 8 patients (22.85%) in the placebo group experienced side effects during the study period, with significantly more side effects in the ZNS 50 mg group (*p* = 0.003). There was no significant difference in side effects between the groups, except for higher sedation (*p* = 0.01) in the ZNS 50 mg and 25 mg groups than in the placebo group (*p* = 0.002 and 0.043, respectively) (Supplementary Table 13).

## Discussion

The current study demonstrated that ZNS was superior to placebo in improving the motor functions (MDS-UPDRS III, the primary endpoint) and motor complications (MDS-UPDRS-IV) of patients with advanced PD, particularly the functional impact of motor fluctuation and painful “Off”-state dystonia and total and motor part MDS-UPDRS scores. On the other hand, there was no change in dyskinesia, NMSs or patients’ QoL.

The current study confirmed the beneficial effect of ZNS on motor functions with some differences. Both ZNS 25 mg and 50 mg demonstrated significant improvement of MDS-UPDRS total, part III and part IV; bradykinesia; and tremor “On” scores compared to baseline, while changes with ZNS 25 mg were more prominent and more significant compared to placebo. Consistently, a previous RCT by Murata et al. showed significant improvement in UPDRS III scores within the ZNS 25 mg and 50 mg groups compared to placebo, with more improvement among the ZNS 25 mg group [7, 8]. Murata et al. reported significant improvement of UPDRS III with ZNS 25 mg, rather than 50 mg [7]. Consistently, lower doses of ZNS increased dopamine and its metabolites in the rat striatum, while higher doses decreased the striatal levels of these substances [24].

On the other hand, in a different study, there was no change in UPDRS III scores when comparing ZNS 25 mg, ZNS 50 mg and placebo, which was attributed to low baseline “On” scores [7]. Therefore, differences between our study and previous reports could be attributed to younger age, shorter duration, lower disease severity and the smaller number of PD patients in our cohort. Population-related differences might have a role, specifically with the recently reported association of the ZNS effect with patients’ genotype [12].

“Off” time reduction is the main outcome of adjunctive therapies and enzyme inhibitors to levodopa in advanced PD [12]. The current study showed significant changes in total MDS-UPDRS part IV scores and in the functional impact of fluctuation subscores from baseline in the ZNS 25 group compared to the placebo group at the 3-month follow-up. Similarly, a 3-month open-label pilot study by Suzuki et al. showed improved total and parts of MDS-UPDRS part IV and found a significant reduction by 1.3 points from baseline. However, this study included only 20 PD patients who received 25 mg (60%) and 50 mg (40%) and did not include a placebo arm [25]. In the previous 2 trials conducted in Japan, no significant change was found between ZNS treatment groups (25, 50 and 100 mg) and placebo with respect to changes from baseline in UPDRS part IV score [7, 8]. These 2 studies used UPDRS, which does not include a rating of the functional impact of fluctuations in part IV.

In previous studies, ZNS 50 mg significantly reduced daily “Off” time compared to placebo as assessed by patients’ diaries. The reduction in daily “Off” time was –0.719 ± 0.179 h/day [7] and 1.395 h/day [8]. In this study, we could not assess changes in daily “Off” time by patient diaries, as there was a high number of dropped-out data. Most of our patients could not complete their diaries, which might be attributed to the illiteracy and low education level of most of the participants. Despite the wide use of patients’ diaries, common problems are associated with obtaining accurate diaries, including the need for proper training, recall bias, reduced compliance, missed data and fatigue diaries in PD clinical trials [26, 27]. These problems are more expected in low-educated and illiterate patients, implying the need for more objective assessment tools of PD long-term complications, such as wearable technology [27]. Therefore, we opted to use the MDS-UPDRS part IV scale.

Few medications showed favourable effects on PD tremor in addition to levodopa. ZNS has been investigated for the treatment of PD rest tremor and other non-PD tremors, such as essential tremor and Holme’s tremor [10]. The potential anti-tremor effect of ZNS has been considered due to its mechanism of the blockade of T-type calcium channels [28]. Interestingly, ZNS 25 mg showed significant improvement in MDS-UPDRS tremor scores, particularly postural tremor, compared to placebo at the 1- and 3-month follow-ups. Consistently, Murata and colleagues reported a significant reduction in UPDRS tremor item 21 scores (action or postural tremors of the hands, but not rest tremor) in ZNS 50 mg compared to placebo [7]. A pilot study reported improvement of rest tremor in 7 out of 9 PD patients with ZNS 100 mg [29]. A recent randomized, placebo-controlled, double-blind study examined the effect of ZNS 25 mg on tremor in 19 tremor-dominant PD patients compared to 18 patients in the placebo group and showed a positive trend of UPDRS tremor score [30].

Another important finding is the improvement of painful “Off” state dystonia with ZNS 50 mg, implying another potential use of ZNS. ZNS was reported as an effective and safe treatment for myoclonus dystonia [31]. Reduction of “Off” time in addition to other mechanisms, such as sodium and calcium channel blocking and GABAergic and glutamatergic modulation, might explain this favourable effect.

Remarkably, the current study confirmed the benefit of ZNS on motor scores without inducing or exacerbating dyskinesia, as assessed by the MDS-UDysRS in addition to MDS-UPDRS part IV. The MDS-UDysRS gives a more objective evaluation of dyskinesia, as it includes an objective examination part rather than the three questions about dyskinesia in UPDRS part IV. The current findings are consistent with previous randomized controlled trials that showed no significant changes in UPDRS part IV between ZNS and placebo groups [7, 8].

Despite the possible nonmotor side effects of ZNS and reported possible beneficial effects on NMSs, few studies have assessed the impact of ZNS on NMSs in PD [10]. The current study comprehensively investigated the NMSs using the NMSS, BDI and HAM-A, in addition to the MDS-UPDRS part I. It showed no worsening or improvement of NMSs using the NMSS compared to the placebo. Worsening of the gastrointestinal tract and mood/cognition subscores of the NMSS were detected within the ZNS 50 mg group at follow-up, which is consistent with its known gastrointestinal side effects [32].

Similarly, previous studies showed no significant change in NMSs using the UPDRS part I with ZNS [7, 8]. Likewise, Suzuki et al. reported no significant change in BDI in an open-label study. However, they reported a beneficial effect of ZNS on sleep using the PD sleep scale −2 and MDS-UPDRS part I [25]. Another recent open-label study of 6 PD patients demonstrated improvement of some sleep parameters of polysomnography, but not the sleep and cognitive scales [11]. Further randomized controlled studies are warranted with a larger number of patients to confirm the impact of ZNS on NMSs in people with PD. Recently, a randomized controlled study aiming to verify the safety and efficacy of ZNS on sleep problems in people with PD was launched [33]. Few studies have examined the impact of ZNS on the QoL of PD patients. The current study demonstrated no significant impact of ZNS on QoL, similar to a previous RCT conducted in Japan [7].

Although the incidence of side effects was higher in the ZNS 25 and 50 mg groups than in the placebo group, hallucinations and worsening or emergence of dyskinesia were similar in the ZNS treatment groups and placebo group. These specific side effects, which are the main concern with dopaminergic medications, are not avoidable in most circumstances with dopaminergic medications and may cause significant disability to some patients. ZNS has both dopaminergic and nondopaminergic mechanisms of action, which may explain the low incidence of hallucinations and dyskinesias; however, longer duration studies may be warranted to assess whether dyskinesias may emerge or worsen with long-term treatment with ZNS.

To the best of our knowledge, this is the first randomized placebo-controlled study outside Japan that investigated the impact of ZNS 25 mg and 50 mg in advanced PD. We used the MDS-UPDRS, including part IV for motor complications, and the MDS-UDysRS for proper assessment of dyskinesia. Moreover, we comprehensively investigated the effect of ZNS on NMSs and QoL using their comprehensive scales. The main limitations of the study are the low number of different groups and the lack of patients’ diaries. Therefore, studies with a larger number of patients with accurate diaries through proper patient training or the use of recent wearable technology are needed.

The current study confirms the favourable effect of ZNS 25 in patients wearing it off as adjunctive therapy with other dopaminergic drugs and its safe profile with no exacerbation of dyskinesia. It showed its beneficial effect on total motor scores and subscores, particularly tremor. Moreover, it demonstrated the limited impact of ZNS on the NMSs and QoL of people with PD.

### Supplementary Information

Below is the link to the electronic supplementary material.

Supplementary file1 (DOCX 94 KB)

## Data Availability

The data and materials used along the current study are available from the corresponding author on reasonable request. No datasets were generated or analysed during the current study.
