# Supplementary material for: Role of zonisamide in advanced Parkinson’s disease: a randomized placebo-controlled study
Source: Neurol Sci. 2024 Feb 20;45(4):1725–34. doi: 10.1007/s10072-024-07396-w (PMC10943138; doi:10.1007/s10072-024-07396-w)
Supplement: Supplementary file 1 — Supplementary file1 (DOCX 94 KB) [file 10072_2024_7396_MOESM1_ESM.docx]

**Supplementary Tables**

**Supplementary table 1 : causes of drug discontinuation among the three groups**

| **Placebo**  **(no=35)** | **ZNS 50mg**  **(no=26)** | **ZNS 25mg**  **(no=34)** |  |
| --- | --- | --- | --- |
| 6 (17.14%) | 5 (19.23%) | 6 (17.64%) | **Patients’ withdrawal** |
| 5 (14.28%) | 0 | 4 (11.76%) | **Protocol violation** |
| 2 (5.71%) | 3 (11.53%) | 3 (8.82%) | **Adverse effects** |
| 1 (2.85%) | 2 (7.69%) | 3 (8.82%) | - **Sedation** |
| 1 (2.85%) | 1 (3.84%) | 1 (2.94%) | - **Gastric irritation** |
| 0 | 0 | 1 (2.94%) | - **Hallucinations** |
| 1 (2.85%) | 1 (3.84%) | 1 (2.94%) | **PD exacerbation** |

**ZNS,** Zonisamide

**Supplementary table 2: baseline demographics and clinical characteristics of the three groups.**

|  | | **ZNS 25 mg**  **(no.=25)** | **ZNS 50 mg**  **(no.=18)** | **placebo**  **(no.=26)** | **P value** |
| --- | --- | --- | --- | --- | --- |
| **Patients receiving levodopa** | | 25 (100%) | 18 (100%) | 26 (100%) |  |
| **Patients receiving dopamine agonists** | | 16 (64%) | 17 (94.4%) | 22 (84.6%) | 0.037* |
| **Patients receiving anticholinergics** | | 12 (48%) | 5 (27.8%) | 9 (34.6%) | 0.370 |
| **Patients receiving amantadine** | | 14 (58.3%) | 12 (66.7%) | 16 (61.5%) | 0.859 |
| **Patients receiving beta blockers** | | 6 (24%) | 6 (33.3%) | 6 (23.1%) | 0.716 |
| **Patients with dyskinesia at baseline** | | 14 (56%) | 9 (50%) | 17 (65.4%) | 0.578 |
| **Years of education** | | 6 (9) | 7.50 (15) | 6 (10) | 0.62 |
| **Education** | **Illiterate** | 9 (36 %) | 6 (33.3 %) | 7 (26.9 %) | 0.732 |
|  | **Read and write** | 2 (8 %) | 1 (5.6 %) | 5 (19.2 %) |  |
|  | **primary school** | 4 (16 %) | 2 (11.1 %) | 3 (11.5 %) |  |
|  | **preparatory school** | 6 (24 %) | 2 (11.1 %) | 5 (19.2 %) |  |
|  | **High school** | 2 (8 %) | 2 (11.1 %) | 1 (3.9 %) |  |
|  | **University graduated** | 2 (8 %) | 5 (27.8 %) | 5 (19.2 %) |  |
| **Functioning** | **Nonfunctioning** | 14 (56 %) | 9 (50 %) | 11 (42.3 %) | 0.533 |
|  | **Functioning** | 5 (20 %) | 7 (38.9 %) | 10 (38.5 %) |  |
|  | **functioning and retired** | 6 (24 %) | 2 (11.1 %) | 5 (19.2 %) |  |
| **Number of Risk Factors** | ***0*** | 18 (72 %) | 15 (83.3) | 22 (84.6 %) | 0.394 |
|  | ***1*** | 4 (16 %) | 2 (11.1 %) | 3 (11.5 %) |  |
|  | ***2*** | 3 (12 %) | 0 (0 %) | 1 (3.9 %) |  |
|  | ***3*** | 0 (0 %) | 1 (5.6 %) | 0 (0 %) |  |
| **Smoking** | | 3 (12 %) | 3 (16.7 %) | 4 (15.4 %) | 0.9 |
| **Substance abuse** | | 0 (0 %) | 2 (11.8 %) | 1 (3.9 %) | 0.187 |
| **DM** | | 5 (20 %) | 1 (5.6 %) | 3 (11.5 %) | 0.366 |
| **HTN** | | 4 (16%) | 1 (5.6 %) | 0 (0 %) | 0.084 |
| **HCV** | | 1 (4 %) | 0 (0 %) | 0 (0 %) | 0.409 |
| **Cardiac illness** | | 0 (0 %) | 2 (11.1 %) | 1 (3.9 %) | 0.209 |
| **Hyperlipidemia** | | 1 (4 % | 1 (5.6 %) | 1 (3.9 %) | 0.958 |
| **Consanguinity** | ***No*** | 20 (80 %) | 11 (61.1 %) | 17 (65.4 %) | 0.349 |
|  | ***Yes*** | 5 (20 %) | 7 (38.9 %) | 9 (34.6 %) |  |
| **Family history of neuropsychiatric disorders** | | 6 (24 %) | 7 (38.9 %) | 8 (30.8 %) | 0.578 |
| **Family history of Parkinsonism** | | 6 (24 %) | 6 (33.3 %) | 8 (30.8 %) | 0.776 |

Data are shown as median (IQR) ornumber (%).

**DM:** Diabetes Mellitus, **HTN:** Hypertension, **HCV:** Hepatitis C virus**, ZNS**: Zonisamide

**p value is significant if <0.*05

**Supplementary table 3: Comparison of baseline motor charachteristics between the three groups.**

|  | **ZNS 25 mg**  **(no.=25)** | **ZNS 50 mg**  **(no.=18)** | **placebo**  **(no.=26)** | **Kruskal-Wallis H** | |
| --- | --- | --- | --- | --- | --- |
|  |  |  |  | **K** | **P value** |
| **Time Up and Go test OFF** | 18.27 (13.59) | 15.80 (16.26) | 15.25 (19.08) | 0.611 | 0.737 |
| **Time Up and Go test ON** | 13.20 (3.95) | 12.16 (13.28) | 11.71 (5.19) | 1.261 | 0.532 |
| **(10- M WT) Comfortable speed OFF** | 0.67 (0.45) | 0.73 (0.44) | 0.72 (0.53) | 0.534 | 0.766 |
| **(10- M WT) Maximum speed OFF** | 0.92 (0.53) | 0.93 (0.61) | 1.01 (0.72) | 0.815 | 0.665 |
| **(10- M WT) Comfortable speed ON** | 0.87 (0.34) | 0.93 (0.31) | 0.89 (0.38) | 0.454 | 0.797 |
| **(10- M WT) Maximum speed ON** | 1.08 (0.43) | 1.12 (0.52) | 1.18 (0.57) | 0.666 | 0.717 |
| **BBS OFF** | 49 (11) | 44.50 (16) | 43.50 (16) | 1.346 | 0.51 |
| **BBS ON** | 51 (8) | 51.50 (9) | 51 (7) | 0.504 | 0.777 |
| **NFOG OFF** | 18 (20) | 23 (13) | 16 (18) | 1.934 | 0.38 |
| **NFOG ON** | 0 (12) | 12(19) | 0 (10) | 3.929 | 0.14 |
| **MDS-UPDRS TS OFF** | 127 (36) | 116.50 (63) | 120.50 (51) | 0.406 | 0.816 |
| **MDS-UPDRS TS ON** | 91 (37) | 84.50 (53) | 87 (37) | 1.25 | 0.535 |
| **MDS-UPDRS Part I (nM-EDL)** | 20 (11) | 17 (12) | 16.50 (7) | 0.85 | 0.654 |
| **MDS-UPDRS Part II (M-EDL)** | 29 (11) | 30.5 (10) | 27.5 (14) | 0.086 | 0.958 |
| **MDS-UPDRS Part III OFF** | 65 (22) | 59 (35) | 65 (15) | 1.848 | 0.397 |
| **MDS-UPDRS Part III ON** | 33 (22) | 30 (17) | 31 (15) | 2.56 | 0.278 |
| Rigidity OFF | 12 (4) | 11.50 (5) | 12 (4) | 1.054 | 0.59 |
| Rigidity ON | 7 (5) | 7 (5) | 7 (3) | 0.504 | 0.777 |
| Bradykinesia OFF | 26 (8) | 23.50 (14) | 25 (8) | 0.946 | 0.623 |
| Bradykinesia ON | 14 (8) | 11.50 (9) | 11.50 (6) | 1.968 | 0.374 |
| PIGD OFF | 10 (5) | 10.50 (6) | 8 (9) | 0.63 | 0.73 |
| PIGD ON | 6 (4) | 7 (5) | 5 (4) | 1.098 | 0.578 |
| Axial OFF | 20 (8) | 21 (10) | 18.50 (10) | 0.31 | 0.856 |
| Axial ON | 11 (10) | 11 (9) | 8 (5) | 0.929 | 0.628 |
| **Tremors OFF** | 13 (13) | 7 (12) | 12.50 (16) | 1.957 | 0.376 |
| Postural tremors OFF | 3 (3) | 1 (2.5) | 2 (4) | 2.970 | 0.227 |
| Kinetic tremors OFF | 2 (2) | 1 (2) | 1.5 (3) | 0.893 | 0.640 |
| Rest tremors OFF | 4 (6) | 2 (5.5) | 4 (7) | 0.840 | 0.657 |
| **Tremors ON** | 7 (9) | 1.50 (5) | 3 (8) | 5.54 | 0.063 |
| Postural tremors ON | 1 (2) | 0 (0) | 0 (1) | 7.418 | **0.025*** |
| Kinetic tremors ON | 1 (1) | 0 (0.5) | 0 (1) | 3.541 | 0.170 |
| Rest tremors ON | 1 (3.5) | 0 (0) | 0 (1.5) | 6.615 | **0.037*** |
| **Modified H&Y** **OFF** | 3 (1.0) | 3 (1.0) | 3 (1.0) | 0.033 | 0.984 |
| **Modified H&Y** **ON** | 2.50 (0.3) | 2.50 (0.0) | 2.50 (0.0) | 0.264 | 0.876 |
| **S&E- ADL-** **OFF** | 50 (25) | 50 (25) | 50 (23) | 0.362 | 0.834 |
| **S&E ADL-** **ON** | 80 (25) | 80 (23) | 80 (10) | 0.653 | 0.722 |
| **MDS-UPDRS Part IV** | 9 (4) | 8 (9) | 9 (7) | 0.164 | 0.921 |
| Time spent with Dyskinesia | 0 (2) | 0 (2) | 1 (2) | 0.692 | 0.708 |
| Functional impact of dyskinesia | 0 (1) | 0 (2) | 0 (1) | 0.688 | 0.709 |
| Time spent in off state | 2 (1) | 3 (1) | 2.50 (1) | 0.204 | 0.903 |
| Functional impact of fluctuations | 3 (1) | 3 (1) | 3 (1) | 0.199 | 0.905 |
| Complexity of motor fluctuations | 2 (1) | 3 (1) | 2.50 (1) | 0.362 | 0.835 |
| Painful off state dystonia | 0 (2) | 0 (2) | 0 (2) | 0.766 | 0.682 |
| **MDS-UDysRS** | 6 (21) | 1.50 (28) | 11.50 (22) | 1.038 | 0.595 |
| Historical dyskinesia Subscore | 4 (14) | 0.50 (20) | 8 (16) | 0.958 | 0.619 |
| Objective dyskinesia Subscore | 0 (7) | 0 (8) | 5.50 (9) | 0.716 | 0.699 |

Data are shown as median, (IQR).

**10-MWT:** 10 meter walking test, **BBS:** Berg Balance Scale, **NFOG:** New freezing of gait questionnaire, **MDS-UPDRS:** Movement Disorder society-unified Parkinson disease rating scale, **TS:** total score, **nM-EDL:** non motor aspects of experiences of daily living, **M-EDL:** motor aspects of experiences of daily living, **PIGD:** Postural instability and gait disorder, **H &Y:** Hoehn & Yahr scale, **S&E-ADL:** Schwab & England-activities of daily living, **MDS-UDysRS:** Movement disorder Society- Unified Dyskinesia Rating scale, **ZNS:** Zonisamide.

**p value is significant if <0.*05.

**Supplementary table 4: Comparison of baseline non motor, cognitive and quality of life charachteristics between the three groups** .

|  | **ZNS 25 mg**  **(no.=25)** | **ZNS 50 mg**  **(no.=18)** | **placebo**  **(no.=26)** | **Kruskal-Wallis H** | |
| --- | --- | --- | --- | --- | --- |
|  |  |  |  | **K** | **P value** |
| **MMSE** | 27 (5) | 27.50 (4) | 28.50 (3) | 1.591 | 0.451 |
| **NMSS** | 56 (48) | 47.50 (30) | 55.50 (33) | 1.03 | 0.597 |
| 1-Cardiovascular | 2 (4) | 1.50 (3) | 2 (2) | 1.486 | 0.476 |
| 2-Sleep/fatigue | 13 (7) | 10 (8) | 10.50 (10) | 1.532 | 0.465 |
| 3-Mood/cognition | 11 (9) | 9.50 (7) | 10 (13) | 0.281 | 0.869 |
| 4-Perceptual problems/Hallucinations | 0 (2) | 0 (0) | 0 (1) | 1.473 | 0.479 |
| 5-Attention/memory | 6 (7) | 6.50 (9) | 6 (7) | 0.156 | 0.925 |
| 6- Gastrointestinal tract | 8 (7) | 5 (5) | 6 (5) | 5.341 | 0.069 |
| 7-Urinary | 5 (8) | 5.50 (6) | 3.50 (8) | 0.7 | 0.705 |
| 8- Sexual function | 4 (3) | 3.00 (2) | 4 (2) | 3.095 | 0.213 |
| 9-Miscellaneous | 6 (8) | 6.50 (8) | 5.50 (6) | 0.448 | 0.799 |
| **BDI** | 19 (11) | 17 (12) | 17.50 (10) | 1.523 | 0.467 |
| **HAM-A** | 15 (10) | 16.50 (12) | 16.50 (8) | 0.596 | 0.742 |
| **PDQ 39 total** | 46.61 (17.55) | 37.14 (26.07) | 39.51 (26.21) | 1.201 | 0.549 |
| Mobility | 60 (47.50) | 48.75 (47.50) | 48.75 (45.63) | 0.432 | 0.806 |
| ADL | 62.50 (37.50) | 50 (52.08) | 54.17 (33.33) | 1.314 | 0.518 |
| Emotional wellbeing | 37.50 (18.75) | 41.67 (30.21) | 33.33 (25.00) | 3.785 | 0.151 |
| Stigma | 62.50 (37.50) | 53.13 (40.63) | 53.13 (34.38) | 1.072 | 0.585 |
| Social support | 16.67 (33.33) | 16.67 (27.08) | 8.33 (25.00) | 0.711 | 0.701 |
| Cognition | 37.50 (21.88) | 28.13 (28.13) | 34.3 (18.75) | 2.363 | 0.307 |
| Communication | 41.67 (29.17) | 37.50 (35.42) | 41.67N(33.33) | 0.667 | 0.716 |
| Bodily discomfort | 50 (29.17) | 37.50 (50.00) | 41.67 (18.75) | 1.562 | 0.458 |

Data are shown as median, (IQR).

**MMSE:** Mini mental state examination, **NMSS:** non motor symptoms scale, **BDI:** Beck Depression Inventory, **HAM-A:** Hamilton anxiety rating scale, **PDQ-39:** Parkinson disease Questionnaire-39, **ADL:** Activities of daily living, **ZNS:** Zonisamide

**Supplementary table 5: Changes in motor, dyskinesia, gait and balance at 1 and 3 months follow-up among ZNS 25 mg group.**

| **ZNS 25 mg group** | **Baseline** | **1-month follow-up** | **3 months follow-up** | **Friedman**  **test** | **Baseline vs 1month** | **Baseline vs**  **3 month** | **1 month vs**  **3 month** |
| --- | --- | --- | --- | --- | --- | --- | --- |
|  |  |  |  |  | **P value** | **P value** | **P value** |
| **MDS-UPDRS TS OFF** | 121 (43) | 110.50 (43) | 106 (49) | 0.009* | 0.027* | 0.027* | 1 |
| **MDS-UPDRS TS ON** | 90.50 (41) | 77.50 (41) | 75 (39) | 0.001* | 0.008* | 0.002* | 1 |
| **MDS-UPDRS Part I (nM_EDL)** | 20 (10) | 19.50 (9) | 19.00 (9) | 0.943 |  | | |
| **MDS-UPDRS Part II (M_EDL)** | 26 (13) | 25.50 (14) | 24.50 (13) | 0.113 |  | | |
| **MDS-UPDRS Part III OFF** | 65 (20) | 62.50 (23) | 58.50 (25) | 0.119 |  | | |
| **MDS-UPDRS Part III ON** | 32.50 (22) | 31 (21) | 29.50 (23) | 0.001* | 0.022* | 0.002* | 1 |
| Rigidity OFF | 12 (4) | 11.50 (4) | 11 (3) | 0.375 |  | | |
| Rigidity ON | 6.50 (5) | 6 (5) | 5 (3) | 0.114 |  | | |
| Bradykinesia OFF | 25.50 (6) | 25 (7) | 23.50 (8) | 0.268 |  | | |
| Bradykinesia ON | 14 (9) | 12(6) | 11.50 (11) | 0.002* | 0.098 | 0.003* | 0.707 |
| **Tremors OFF** | 13 (14) | 9 (14) | 8.50 (16) | 0.044* | 0.173 | 0.098 | 1 |
| Postural tremors OFF | 3 (3) | 2 (2) | 2 (3) | 0.004* | 0.007* | 0.012* | 0.234 |
| Kinetic tremors OFF | 2 (2) | 1 (1) | 1 (3) | 0.032* | 0.317 | 0.039 | 0.059 |
| Rest tremors OFF | 4 (6) | 2.5 (5.75) | 3 (7) | 0.704 |  |  |  |
| **Tremors ON** | 7 (10) | 5.50 (10) | 4.50 (10) | 0.001* | 0.013* | 0.027* | 1 |
| Postural tremors ON | 1 (2) | 0.5 (1.75) | 1 (2) | 0.006* | 0.007* | 0.008* | 0.480 |
| Kinetic tremors ON | 1 (1) | 0 (1) | 0 (1) | 0.197 |  |  |  |
| Rest tremors ON | 1 (3.5) | 1 (2.75) | 1 (4) | 0.256 |  |  |  |
| **MDS-UPDRS Part IV** | 8.50 (4) | 8.50 (4) | 8 (4) | 0.135 |  | | |
| Time spent with Dyskinesia | 0 (1) | 0 (1) | 0 (1) | 0.368 |  | | |
| Functional impact of dyskinesia | 0 (1) | 0 (1) | 0 (1) | 0.607 |  | | |
| Time spent in off state | 2 (1) | 2 (1) | 2 (1) | 0.247 |  | | |
| Functional impact of fluctuations | 3 (1) | 2 (1) | 2 (1) | 0.032* | 0.707 | 0.404 | 1 |
| Complexity of motor fluctuations | 2 (1) | 2 (1) | 2 (1) | 0.321 |  | | |
| Painful off state dystonia | 0 (1) | 0 (1) | 0 (2) | 0.368 |  | | |
| **Modified H&Y** OFF | 3 (0.8) | 3 (0.8) | 3 (0) | 1 |  | | |
| **Modified H&Y** ON | 2.5 (0) | 2.5 (0) | 2.5 (0) | 0.368 |  | | |
| **S&E- ADL** OFF | 50 (20) | 50 (20) | 50 (18) | 0.177 |  | | |
| **S&E-ADL** ON | 80 (25) | 80 (25) | 80 (28) | 0.678 |  | | |
| **MDS-UDysRS** | 5.50 (19) | 6.50 (22) | 6 (19) | 0.059 |  | | |
| Historical dyskinesia Subscore | 4 (13) | 6.50 (15) | 5 (11) | 0.019* | 0.291 | 1 | 0.173 |
| Objective dyskinesia Subscore | 0 (7) | 0 (7) | 0 (7) | 0.747 |  | | |
| **Time Up and Go test OFF** | 18.11 (13.11) | 18.30 (12.36) | 17.60 (11.98) | 0.382 |  | | |
| **Time Up and Go test ON** | 12.53 (3.34) | 12.49 (4.89) | 12.61 (4.93) | 0.549 |  | | |
| **(10- M WT) Comfortable speed OFF** | 0.67 (0.45) | 0.58 (0.51) | 0.67 (0.42) | 0.387 |  | | |
| **(10- M WT) Maximum speed OFF** | 0.95 (0.54) | 0.82 (0.54) | 0.92 (0.43) | 0.387 |  | | |
| **(10- M WT) Comfortable speed ON** | 0.89 (0.35) | 0.82 (0.28) | 0.85 (0.30) | 0.705 |  | | |
| **(10- M WT) Maximum speed ON** | 1.09 (0.43) | 1.02 (0.30) | 1.08 (0.27) | **0.043*** | 0.173 | 1 | 0.053 |
| **BBS OFF** | 43.50 (12) | 44.50 (13) | 45 (12) | 0.495 |  | | |
| **BBS ON** | 51 (8) | 51.50 (9) | 50.50 (9) | 0.379 |  | | |
| **NFOG OFF** | 17.50 (13) | 18 (10) | 17.50 (12) | 0.442 |  | | |
| **NFOG ON** | 0 (12) | 0 (13) | 0 (11) | 0.236 |  | | |

Data are shown as median, (IQR).

**MDS-UPDRS:**Movement Disorder society-unified Parkinson disease rating scale, **TS:** total score, **nM-EDL:** non motor aspects of experiences of daily living, **M-EDL:** motor aspects of experiences of daily living, **H&Y:**Hoehn & Yahr scale, **S&E-ADL:** Schwab & England-activities of daily living**, MDS-UDysRS:** Movement disorder Society- Unified Dyskinesia Rating scale , **10-MWT:** 10 meter walking test, **BBS:** Berg Balance Scale, **NFOG:**New freezing of gait questionnaire, **ZNS:** Zonisamide

******p value*** *is significant if <0.*05.

**Supplementary table 6: changes in non-motor symptoms and quality of life at 1 and 3 months follow-up among ZNS 25 mg group.**

| **ZNS 25 mg group** | **Baseline** | **1-month follow-up** | **3 months follow-up** | **Friedman**  **test** | **Baseline vs 1month** | **Baseline vs**  **3 month** | **1 month vs**  **3 month** |
| --- | --- | --- | --- | --- | --- | --- | --- |
|  |  |  |  |  | **P value** | **P value** | **P value** |
| **NMSS** | 54.00 (44) | 52.50 (45) | 51.50 (52) | 0.911 |  | | |
| 1-Cardiovascular | 2 (4) | 2 (3) | 2 (3) | 0.549 |  | | |
| 2-Sleep/fatigue | 10.50 (8) | 10.50 (9) | 10.50 (9) | 0.654 |  | | |
| 3-Mood/ cognition | 10.50 (8) | 10.50 (8) | 10.50 (10) | 0.331 |  | | |
| 4-Perceptual problems/Hallucinations | 0 (2) | 1 (2) | 1 (2) | 0.024* | 1 | 0.805 | 1 |
| 5-Attention/ memory | 5 (5) | 6 (5) | 6 (6) | 0.071 |  | | |
| 6- Gastrointestinal tract | 7.50 (6) | 6 (7) | 7 (8) | 0.796 |  | | |
| 7-Urinary | 4.50 (10) | 4 (9) | 4.50 (9) | 0.944 |  | | |
| 8- Sexual function | 4 (2) | 4 (3) | 4 (1) | 0.289 |  | | |
| 9-Miscellaneous | 5.50 (7) | 6 (6) | 6 (4) | 0.936 |  | | |
| **BDI** | 19 (10) | 20 (12) | 19 (11) | 0.710 |  | | |
| **HAM-A** | 16.00 (9) | 16.50 (7) | 15.50 (6) | 0.850 |  | | |
| **PDQ 39 total** | 46.85 (23.06) | 41.98 (19.66) | 42.27 (20.30) | 0.247 |  | | |
| Mobility | 55 (43.13) | 50 (42.50) | 47.50 (46.25) | 0.110 |  | | |
| ADL | 54.17 (35.42) | 45.83 (33.33) | 52.08 (37.50) | 0.321 |  | | |
| Emotional wellbeing | 37.50 (18.75) | 37.50 (17.71) | 31.25 (26.04) | 0.205 |  | | |
| Stigma | 65.63 (34.38) | 50 (31.25) | 50 (31.25) | 0.037* | 0.342 | 0.081 | 1 |
| Social support | 16.67 (33.33) | 12.50 (25) | 8.33 (25) | 0.191 |  | | |
| Cognition | 34.38 (23.44) | 37.50 (18.75) | 37.50 (17.19) | 0.070 |  | | |
| Communication | 33.33 (31.25) | 33.33 (25) | 33.33 (25) | 0.381 |  | | |
| Bodily discomfort | 45.83 (25) | 41.67 (14.58) | 41.67 (22.92) | 0.882 |  | | |

Data are shown as median, (IQR).

**NMSS;** non motor symptoms scale, **BDI;** Beck Depression Inventory, **HAM-A;** Hamilton anxiety rating scale, **PDQ-39**: Parkinson disease Questionnaire-39, **ADL:** activities of daily living, **ZNS**: Zonisamide

**p value is significant if <0.*05.

**Supplementary table 7: Changes in motor, dyskinesia, gait and balance at 1 and 3 months follow-up among ZNS 50 mg group.**

| **ZNS 50 mg group** | **Baseline** | **1-month follow-up** | **3 months follow-up** | **Friedman**  **test** | **Baseline vs 1month** | **Baseline vs**  **3 month** | **1 month vs**  **3 month** |
| --- | --- | --- | --- | --- | --- | --- | --- |
|  |  |  |  |  | **P value** | **P value** | **P value** |
| **MDS-UPDRS TS OFF** | 116 (66) | 106 (54) | 105 (54) | 0.010* | 0.146 | 0.011* | 1 |
| **MDS-UPDRS TS ON** | 85 (54) | 85 (47) | 88 (45) | 0.051 |  | | |
| **MDS-UPDRS Part I (nM_EDL)** | 15 (12) | 17 (10) | 17 (11) | 0.414 |  | | |
| **MDS-UPDRS Part II (M_EDL)** | 29 (12) | 25 (12) | 27 (15) | 0.032* | 0.077 | 0.096 | 1 |
| **MDS-UPDRS Part III OFF** | 61 (37) | 60 (29) | 59 (29) | 0.003* | 0.510 | 0.003* | 0.178 |
| **MDS-UPDRS Part III ON** | 31 (16) | 27 (22) | 24 (23) | 0.125 |  | | |
| Rigidity OFF | 12 (5) | 11 (5) | 10 (5) | 0.019* | 1 | 0.049* | 0.435 |
| Rigidity ON | 7 (6) | 6 (5) | 5 (5) | 0.790 |  | | |
| Bradykinesia OFF | 24 (14) | 21 (13) | 21 (13) | 0.080 |  | | |
| Bradykinesia ON | 12 (7) | 11 (8) | 11 (9) | 0.032* | 0.077 | 0.178 | 1 |
| PIGD OFF | 10 (7) | 8 (7) | 9 (9) | 0.013* | 0.119 | 0.368 | 1 |
| PIGD ON | 7 (6) | 5 (6) | 6 (6) | 0.225 |  | | |
| Axial OFF | 21 (10) | 20 (9) | 19 (11) | 0.013* | 0.049* | 0.077 | 1 |
| Axial ON | 11 (9) | 11(10) | 10 (8) | 0.945 |  | | |
| **Tremors OFF** | 7 (13) | 7 (13) | 8 (13) | 0.368 |  | | |
| Postural tremors OFF | 1 (2.5) | 1 (1.25) | 1 (3) | 0.264 |  | | |
| Kinetic tremors OFF | 1 (2) | 1 (2) | 1 (2) | 0.125 |  | | |
| Rest tremors OFF | 2 (5.5) | 2 (7.25) | 2 (7) | 0.529 |  | | |
| **Tremors ON** | 2 (5) | 1 (7) | 1 (9) | 0.965 |  | | |
| Postural tremors ON | 0 (0) | 0 (0.25) | 0 (1) | 0.846 |  | | |
| Kinetic tremors ON | 0 (0.5) | 0 (0) | 0 (0.5) | 0.368 |  | | |
| Rest tremors ON | 0 (0) | 0 (1.5) | 0 (1.5) | 0.039* | 0.066 | 0.109 | 0.0317 |
| **MDS-UPDRS Part IV** | 8 (9) | 7 (6) | 7 (8) | 0.001* | 0.005* | 0.012* | 1 |
| Time spent with Dyskinesia | 0 (2) | 0 (2) | 0 (2) | 0.368 |  | | |
| Functional impact of dyskinesia | 0 (2) | 0 (2) | 0 (2) | 0.050 |  | | |
| Time spent in off state | 3 (1) | 2 (1) | 2 (2) | 0.097 |  | | |
| Functional impact of fluctuations | 3 (1) | 2 (1) | 2 (2) | 0.001* | 0.146 | 0.049* | 1 |
| Complexity of motor fluctuations | 3 (1) | 2 (1) | 2 (2) | 0.121 |  | | |
| Painful off state dystonia | 0 (2) | 0 (2) | 0 (2) | 0.018* | 0.910 | 0.910 | 1 |
| **Modified H&Y** OFF | 3 (1.0) | 3 (1.0) | 3 (1.0) | 0.135 |  |  |  |
| **Modified H&Y** ON | 2.5 (0) | 2.5 (0) | 2.5 (0) | 0.717 |  |  |  |
| **S&E-ADL** OFF | 50 (30) | 50 (30) | 50 (25) | 0.257 |  |  |  |
| **S&E- ADL** ON | 80 (25) | 70 (30) | 70 (25) | 0.135 |  |  |  |
| **MDS-UDysRS** | 0 (21) | 5 (23) | 0 (23) | 0.616 |  | | |
| Historical dyskinesia Subscore | 0 (15) | 3 (15) | 0 (15) | 0.641 |  | | |
| Objective dyskinesia Subscore | 0 (8) | 0 (9) | 0 (8) | 0.878 |  | | |
| **Time Up and Go test OFF** | 15.75 (17.47) | 14.96 (16.01) | 14.4150 (16.81) | 0.481 |  | | |
| **Time Up and Go test ON** | 12.16 (14.37) | 11.65 (9.36) | 11.8500 (5.56) | 0.399 |  | | |
| **(10- M WT) Comfortable speed OFF** | 0.67 (0.49) | 0.72 (0.36) | 0.73 (0.57) | 0.327 |  | | |
| **(10- M WT) Maximum speed OFF** | 0.93 (0.73) | 0.97 (0.71) | 1 (0.63) | 0.006 |  | | |
| **(10- M WT) Comfortable speed ON** | 0.91 (0.33) | 0.95 (0.49) | 0.93 (0.42) | 0.193 |  | | |
| **(10- M WT) Maximum speed ON** | 1.11 (0.59) | 1.11 (0.64) | 1.12 (0.59) | 0.174 |  | | |
| **BBS OFF** | 44.50 (19) | 47.50 (17) | 50 (17) | 0.703 |  | | |
| **BBS ON** | 51.50 (9) | 52 (9) | 53 (11) | 0.239 |  | | |
| **NFOG OFF** | 23 (13) | 20.50 (13) | 19 (13) | 0.014* |  | | |
| **NFOG ON** | 13 (20) | 13 (17) | 11 (18) | 0.022* |  | | |

Data are shown as median, (IQR).

**MDS-UPDRS:** Movement Disorder society-unified Parkinson disease rating scale, **TS:** total score, **nM-EDL**: non motor aspects of experiences of daily living, **M-EDL**: motor aspects of experiences of daily living, **PIGD:**Postural instability and gait disorder, **H-Y:** Hoehn & Yahr scale, **S&E-ADL:** Schwab & England- Activities of daily living, **MDS-UDysRS:** Movement disorder Society- Unified Dyskinesia Rating scale , **10-MWT:** 10 meter walking test, **BBS:** Berg Balance Scale, **NFOG:** New freezing of gait questionnaire.

******p value*** *is significant if <0.*05.

**Supplementary table 8: changes in non-motor symptoms and quality of life at 1 and 3 months follow-ups among ZNS 50 mg group.**

| **ZNS 50 mg group** | **Baseline** | **1-month follow-up** | **3 months follow-up** | **Friedman**  **test** | **Baseline vs 1month** | **Baseline vs**  **3 month** | **1 month vs**  **3 month** |
| --- | --- | --- | --- | --- | --- | --- | --- |
|  |  |  |  |  | **P value** | **P value** | **P value** |
| **NMSS** | 43 (25) | 54 (32) | 56 (30) | 0.148 |  | | |
| 1-Cardiovascular | 2 (3) | 1 (4) | 1 (4) | 0.792 |  | | |
| 2-Sleep/fatigue | 10 (8) | 10 (8) | 9 (7) | 0.393 |  | | |
| 3-Mood/ cognition | 9 (5) | 10 (9) | 10 (11) | 0.011* | 0.098 | 0.039* | 1 |
| 4-Perceptual problems/Hallucinations | 0 (0) | 0 (0) | 0 (1) | 0.905 |  | | |
| 5-Attention/ memory | 6 (9) | 7 (10) | 7 (9) | 0.171 |  | | |
| 6- Gastrointestinal tract | 5 (6) | 6 (8) | 5 (7) | 0.005* | 0.030* | 0.119 | 1 |
| 7-Urinary | 5 (5) | 4 (5) | 5 (5) | 0.126 |  | | |
| 8- Sexual function | 3 (2) | 3 (2) | 4 (1) | 0.430 |  | | |
| 9-Miscellaneous | 6 (8) | 6 (7) | 6 (8) | 0.444 |  | | |
| **BDI** | 17 (11) | 19 (6) | 20 (9) | 0.374 |  | | |
| **HAM-A** | 16 (12) | 16 (10) | 16 (9) | 0.586 |  | | |
| **PDQ 39 total** | 37.14 (22.80) | 39.48 (21.47) | 37.47 (27.34) | 0.137 |  | | |
| Mobility | 48.75 (44.38) | 48.75 (53.75) | 48.75 (58.75) | 0.551 |  | | |
| ADL | 50 (56.25) | 52.08 (55.21) | 50 (51.04) | 0.199 |  | | |
| Emotional wellbeing | 41.67 (25) | 33.33 (30.21) | 39.58 (39.58) | 0.418 |  | | |
| Stigma | 53.13 (43.75) | 53.13 (29.69) | 53.13 (35.94) | 0.004* | 0.062 | 0.014* | 1 |
| Social support | 16.67 (25) | 16.67 (25.00) | 20.83 (31.25) | 0.575 |  | | |
| Cognition | 21.88 (34.38) | 18.75 (29.69) | 21.88 (23.44) | 0.862 |  | | |
| Communication | 37.50 (37.50) | 33.33 (22.92) | 33.3 (16.67) | 0.920 |  | | |
| Bodily discomfort | 37.50 (50) | 37.50 (31.25) | 33.33 (31.25) | 0.441 |  | | |

Data are shown as median, (IQR).

**NMSS;** non motor symptoms scale, **BDI;** Beck Depression Inventory, **HAM-A;** Hamilton anxiety rating scale, **PDQ-39:** Parkinson disease Questionnaire-39, **ADL:** activities of daily living, **ZNS:** Zonisamide

**p value is significant if <0.*05.

**Supplementary table 9: Changes in motor, dyskinesia, gait and balance at 1 and 3 months follow-up among placebo group.**

| **Placebo group** | **Baseline** | **1-month follow-up** | **3 months follow-up** | **Friedman**  **test** | **Baseline vs 1month** | | **Baseline vs**  **3 month** | | **1 month vs**  **3 month** | |
| --- | --- | --- | --- | --- | --- | --- | --- | --- | --- | --- |
|  |  |  |  |  | **P value** | | **P value** | | **P value** | |
| **MDS-UPDRS TS OFF** | 123 (48) | 119 (60) | 122 (61) | 0.317 |  | | | | | |
| **MDS-UPDRS TS ON** | 88 (33) | 92 (39) | 90 (44) | 0.776 |  | | | | | |
| **MDS-UPDRS Part I (nM_EDL)** | 17 (7) | 18 (8) | 18 (7) | 0.054 |  | | | | | |
| **MDS-UPDRS Part II (M_EDL)** | 28 (13) | 29 (14) | 27 (14) | 0.546 |  | | | | | |
| **MDS-UPDRS Part III OFF** | 67 (15) | 62 (22) | 64 (24) | 0.121 |  | | | | | |
| **MDS-UPDRS Part III ON** | 31 (15) | 29 (15) | 30 (11) | 0.887 |  | | | | | |
| Rigidity OFF | 12 (3) | 12 (3) | 12 (4) | 0.341 |  | | | | | |
| Rigidity ON | 6 (4) | 7 (2) | 6 (3) | 0.252 |  | | | | | |
| Bradykinesia OFF | 25 (8) | 24 (9) | 24 (11) | 0.316 |  | | | | | |
| Bradykinesia ON | 11 (7) | 10 (6) | 11 (7) | 0.865 |  | | | | | |
| PIGD OFF | 8 (7) | 9 (6) | 9 (8) | 0.766 |  | | | | | |
| PIGD ON | 5 (4) | 6 (5) | 5 (4) | 0.898 |  | | | | | |
| Axial OFF | 19 (9) | 18 (10) | 21 (9) | 0.641 |  | | | | | |
| Axial ON | 8 (5) | 9 (5) | 10 (6) | 0.001* | 1 | 0.005* | | | | 0.026 |
| **Tremors OFF** | 13 (17) | 12 (17) | 13 (17) | 0.45 |  | | | | | |
| Postural tremors OFF | 2 (4) | 2 (3) | 1 (3) | 0.522 |  | | | | | |
| Kinetic tremors OFF | 1.5 (3) | 1.5 (3) | 1 (3) | 0.565 |  | | | | | |
| Rest tremors OFF | 4 (7) | 4 (7) | 4 (8) | 0.572 |  | | | | | |
| **Tremors ON** | 3 (9) | 4 (10) | 3 (11) | 0.569 |  | | | | | |
| Postural tremors ON | 0 (1) | 0 (1.25) | 0 (1.5) | 0.756 |  | | | | | |
| Kinetic tremors ON | 0 (1) | 0 (1) | 0 (1) | 0.846 |  | | | | | |
| Rest tremors ON | 0 (1.5) | 0 (2.25) | 1 (3) | 0.169 |  | | | | | |
| **MDS-UPDRS Part IV** | 9 (5) | 9 (4) | 9 (7) | 0.739 |  | | | | | |
| Time spent with Dyskinesia | 1 (2) | 1 (2) | 1 (2) | 0.779 |  | | | | | |
| Functional impact of dyskinesia | 0 (1) | 0 (1) | 0 (1) | 0.717 |  | | | | | |
| Time spent in off state | 3 (1) | 3 (1) | 3 (1) | 1 |  | | | | | |
| Functional impact of fluctuations | 3 (1) | 3 (1) | 3 (1) | 0.819 |  | | | | | |
| Complexity of motor fluctuations | 3 (1) | 2 (1) | 3 (1) | 0.651 |  | | | | | |
| Painful off state dystonia | 0 (2) | 0 (2) | 0 (2) | 0.050 |  | | | | | |
| **Modified H&Y** OFF | 3 (1.0) | 3 (1.0) | 3 (1.0) | 0.368 |  | | | | | |
| **Modified H&Y** ON | 2.5 (0) | 2.5 (0) | 2.5 (0.3) | 0.368 |  | | | | | |
| **S&E-ADL** OFF | 50 (20) | 50 (20) | 60 (20) | 0.199 |  | | | | | |
| **S&E-ADL** ON | 80 (10) | 80 (20) | 80 (10) | 0.913 |  | | | | | |
| **MDS-UDysRS** | 11 (21) | 13 (25) | 10 (22) | 0.055 |  | | | | | |
| Historical dyskinesia Subscore | 8 (15) | 9 (19) | 8 (14) | 0.035* | 0.161 | | | 1 | 0.368 | |
| Objective dyskinesia Subscore | 5 (8) | 3 (8) | 5 (8) | 0.244 |  | | | | | |
| **Time Up and Go test OFF** | 15.55 (20.45) | 14.54 (18.37) | 14.56 (19.47) | 0.481 |  | | | | | |
| **Time Up and Go test ON** | 12.72 (7.78) | 12.33 (5.94) | 11.53 (6.06) | 0.823 |  | | | | | |
| **(10- M WT) Comfortable speed OFF** | 0.69 (0.51) | 0.78 (0.52) | 0.77 (0.54) | 0.327 |  | | | | | |
| **(10- M WT) Maximum speed OFF** | 1.0 (0.73) | 1.01 (0.73) | 0.98 (0.72) | 0.276 |  | | | | | |
| **(10- M WT) Comfortable speed ON** | 0.85 (0.38) | 0.91 (0.39) | 0.92 (0.40) | 0.467 |  | | | | | |
| **(10- M WT) Maximum speed ON** | 1.07 (0.57) | 1.17 (0.49) | 1.13 (0.58) | 0.276 |  | | | | | |
| **BBS OFF** | 43 (16) | 45 (13) | 45 (14) | 0.316 |  | | | | | |
| **BBS ON** | 51 (6) | 51 (8) | 51 (9) | 0.418 |  | | | | | |
| **NFOG OFF** | 16 (13) | 17 (11) | 19 (13) | 0.127 |  | | | | | |
| **NFOG ON** | 0 (11) | 0 (11) | 0 (12) | 0.809 |  | | | | | |

Data are shown as median, (IQR).

**MDS-UPDRS:** Movement Disorder society-unified Parkinson disease rating scale, **TS:** total score, **nM-EDL:** non motor aspects of experiences of daily living, **M-EDL:** motor aspects of experiences of daily living, **PIGD:** Postural instability and gait disorder, **H&Y:** Hoehn & Yahr scale, **S&E-ADL**: Schwab & England- activities of daily living, **MDS-UDysRS:** Movement disorder Society- Unified Dyskinesia Rating scale, **10-MWT:** 10 meter walking test, **BBS**: Berg Balance Scale, **NFOG:** New freezing of gait questionnaire, **ZNS:** Zonisamide.

******p value*** *is significant if <0.*05.

**Supplementary table 10: changes in non-motor symptoms and quality of life at 1 and 3 months follow-ups among placebo group.**

| **Placebo group** | **Baseline** | **1-month follow-up** | **3 months follow-up** | **Friedman**  **test** | **Baseline vs 1 month** | | **Baseline vs**  **3 month** | **1 month vs**  **3 month** | |
| --- | --- | --- | --- | --- | --- | --- | --- | --- | --- |
|  |  |  |  |  | **P value** | | **P value** | **P value** | |
| **NMSS** | 56 (33) | 54 (35) | 56 (36) | 0.053 |  | | | | |
| 1-Cardiovascular | 2 (2) | 2 (2) | 2 (1) | 0.529 |  | | | | |
| 2-Sleep/fatigue | 10 (8) | 11 (9) | 11 (10) | 0.528 |  | | | | |
| 3-Mood/ cognition | 10 (11) | 10 (11) | 10 (10) | 0.343 |  | | | | |
| 4-Perceptual problems/Hallucinations | 0 (1) | 0 (1) | 0 (1) | 1 |  | | | | |
| 5-Attention/ memory | 6 (7) | 6 (6) | 7 (7) | 0.331 |  | | | | |
| 6- Gastrointestinal tract | 6 (6) | 6 (6) | 6 (7) | 0.028* | 0.092 | 0.368 | | | 1 |
| 7-Urinary | 3 (11) | 4 (9) | 4 (9) | 0.270 |  | | | | |
| 8- Sexual function | 4 (1) | 4 (2) | 4 (3) | 0.223 |  | | | | |
| 9-Miscellaneous | 6 (5) | 6 (6) | 6 (5) | 0.129 |  | | | | |
| **BDI** | 17 (10) | 18 (9) | 19 (10) | 0.463 |  | | | | |
| **HAM-A** | 16 (9) | 15 (8) | 16 (10) | 0.985 |  | | | | |
| **PDQ 39 total** | 40.36 (26.20) | 37.08 (20.96) | 38.39 (23.9) | **0.005*** | 0.004 | | 0.192 | 0.495 | |
| Mobility | 50 (41.25) | 47.50 (42.50) | 47.50 (47.50) | 0.831 |  | | | | |
| ADL | 54.17 (33.33) | 50 (33.33) | 50 (37.50) | **0.031*** | 0.076 | | 0.269 | 1 | |
| Emotional wellbeing | 33.33 (22.92) | 25 (20.83) | 29.17 (27.08) | **0.015*** | 1 | | 0.495 | 0.062 | |
| Stigma | 50 (31.25) | 50 (31.25) | 50 (37.50) | **0.039*** | 0.316 | | 0.112 | 1 | |
| Social support | 8.33 (20.83) | 8.33 (16.67) | 8.33 (20.83) | 0.063 |  | | | | |
| Cognition | 37.50 (15.63) | 31.25 (18.75) | 37.50 (18.75) | **0.046*** | 0.651 | | 1 | 0.135 | |
| Communication | 41.67 (25.00) | 33.33 (20.83) | 41.67 (25.00) | 0.595 |  | | | | |
| Bodily discomfort | 41.67 (29.17) | 41.67 (25) | 41.67 (25.00) | 0.838 |  | | | | |

Data are shown as median, (IQR).

**NMSS;** non motor symptoms scale, **BDI;** Beck Depression Inventory, **HAM-A;** Hamilton anxiety rating scale, **PDQ-39:** Parkinson disease Questionnaire-39, **ADL:** activities of daily living, **ZNS:** Zonisamide.

**p value is significant if <0.*05.

**Supplementary table 11: Comparison of changes of motor, non-motor symptoms and quality of life from baseline at 1 month follow-up among the 3 groups**.

|  | **ZNS 25 mg** | **ZNS 50 mg** | **Placebo** | **Kruskal-Wallis H** |
| --- | --- | --- | --- | --- |
|  | **(no.=25)** | **(no.=18)** | **(no.=26)** | **P value** |
| **Modified H&Y OFF** | 0 (0) | 0 (0) | 0 (.0) | 0.078 |
| **Modified H&Y ON** | 0 (0) | 0 (0) | 0 (0) | 0.444 |
| **S&E ADL OFF** | 0 (0) | 0 (15) | 0 (10) | 0.207 |
| **S&E ADL ON** | 0 (0) | 0 (5) | 0 (0) | 0.186 |
| **PIGD OFF** | 0 (1) | 1 (3) | 0 (1) | 0.318 |
| **PIGD ON** | 0 (1) | 0 (3) | 0 (1) | 0.947 |
| **Axial OFF** | 1 (2) | 1.00 (3) | 0 (2) | 0.543 |
| **Axial ON** | 0 (2) | 0 (3) | 0 (2) | 0.404 |
| **Time spent with Dyskinesia** | 0 (0) | 0 (0) | 0 (0) | 0.078 |
| **Functional impact of dyskinesia** | 0 (0) | 0 (0) | 0 (0) | 0.415 |
| **Time spent in off state** | 0 (0) | 0 (1) | 0 (0) | 0.233 |
| **Functional impact of fluctuations** | 0 (1) | 0 (1) | 0 (0) | 0.132 |
| **Complexity of motor fluctuations** | 0 (0) | 0 (0) | 0 (0) | 0.761 |
| **Time Up and Go test OFF** | 0.53 (2.36) | 0.0 (3.36) | 0.59 (2.09) | 0.477 |
| **Time Up and Go test ON** | 0.21 (1.46) | 0.42 (1.96) | 0.31 (1.47) | 0.652 |
| **10- M WT Comfortable speed OFF** | 0.016 (0.10) | 0.0027 (0.07) | 0.02 (0.08) | 0.681 |
| **10- M WT Maximum speed OFF** | 0.0023 (0.07) | 0.008 (0.08) | 0.0071 (0.08) | 0.752 |
| **BBS OFF** | 0 (4) | 0 (3) | 0.50 (3) | 0.572 |
| **NFOG OFF** | 0 (2) | 2 (4) | 0.50 (2) | 0.092 |
| **NMSS** | -1 (6) | -2 (8) | -2 (4) | 0.457 |
| 1-Cardiovascular | 0 (1) | 0 (1) | 0 (0) | 0.472 |
| 2-Sleep/fatigue | 1 (3) | 0 (4) | 0 (3) | 0.497 |
| 3-Mood/cognition | 0 (4) | -1 (2) | 0 (2) | 0.162 |
| 4-Perceptual problems/ Hallucinations | 0 (0) | 0 (0) | 0 (0) | 0.405 |
| 5-Attention/memory | -1 (1) | 1 (3) | 0 (1) | 0.731 |
| 6- Gastrointestinal tract | -1 (2) | -1 (2) | -1 (1) | 0.522 |
| 7-Urinary | 0 (2) | 0 (2) | -0.50 (1) | 0.439 |
| 8- Sexual function | 0 (1) | 0 (1) | 0 (0) | 0.894 |
| 9-Miscellaneous | 0 (4) | 0 (3) | -1 (1) | 0.074 |
| **BDI** | 0 (5) | 0 (4) | 0 (3) | 0.429 |
| **HAM-A** | 0 (4) | 1 (3) | 0 (2) | 0.722 |
| **PDQ 39 total** | 1.56 (5.81) | 3.13 (7.92) | 1.46 (3.15) | 0.745 |
| Mobility | 0 (7.50) | 2.50 (15.00) | .0 (5.63) | 0.879 |
| ADL | 4.17 (14.58) | 0 (12.50) | 2.08 (9.37) | 0.86 |
| Emotional wellbeing | 0 (8.33) | 0 (8.33) | 0 (5.21) | 0.79 |
| Stigma | 6.25 (12.50) | 6.25 (9.38) | 6.25 (7.81) | 0.756 |
| Social support | 0 (8.33) | 0 (8.33) | 0 (8.33) | 0.731 |
| Cognition | 0 (9.38) | 0 (12.50) | 0 (6.25) | 0.263 |
| Communication | 0 (16.67) | 0 (16.67) | 0 (8.33) | 0.326 |
| Bodily discomfort | 0 (25) | 0 (16.67) | 0 (10.42) | 0.574 |

Data are shown as median, (IQR).

**H&Y:** Hoehn & Yahr scale, **S&E-ADL:** Schwab and England-activities of daily living, **PIGD:** Postural instability and gait disorder, **10-MWT:** 10 meter walking test, **BBS:** Berg Balance Scale, **NFOG:** New freezing of gait questionnaire, **NMSS;** non motor symptoms scale, **BDI;** Beck Depression Inventory, **HAM-A;** Hamilton anxiety rating scale, **PDQ-39:** Parkinson disease questionnaire-39, **ADL:** activities of daily living, **ZNS:** Zonisamide

**Supplementary table 12**: **comparison of changes of motor, non-motor symptoms and quality of life from baseline at 3 months follow-up among the 3 groups.**

|  | **ZNS 25 mg** | **ZNS 50 mg)** | **Placebo** | **Kruskal-Wallis H** |
| --- | --- | --- | --- | --- |
|  | **(no.=20)** | **(no.=17)** | **(no.=21)** | **P value** |
| **Modified H&Y OFF** | 0 (0) | 0 (0) | 0 (0) | 0.498 |
| **Modified H&Y ON** | 0 (0) | 0 (0) | 0 .(0) | 0.460 |
| **S&E ADL OFF** | 0 (10) | 0 (20) | 0 (10) | 0.976 |
| **S&E ADL ON** | 0 (0) | 0 (10) | 0 (0) | 0.877 |
| **PIGD OFF** | 0 (1) | 0 (3) | 0 (2) | 0.268 |
| **PIGD ON** | 0 (1) | 1 (2) | 0 (0) | 0.447 |
| **Axial OFF** | 0.50 (3) | 2 (4) | 0 (4) | 0.309 |
| **Axial ON** | -0.50 (3) | 0 (2) | -1 (3) | 0.118 |
| **Time spent with dyskinesia** | 0 (0) | 0 (0) | 0 (0) | 0.841 |
| **Functional impact of dyskinesia** | 0 (0) | 0 (0) | 0 (0) | 0.329 |
| **Time spent in off state** | 0 (0) | 0 (1) | 0 (0) | 0.13 |
| **Historical dyskinesia Subscore** | 0 (1) | 0 (4) | 0 (3) | 0.532 |
| **Objective dyskinesia Subscore** | 0 (0) | 0 (0) | 0 (1) | 0.397 |
| **Time Up and Go test OFF** | 0.52 (3.18) | 0.77 (3.93) | 0.7800 (3.13) | 0.736 |
| **Time Up and Go test ON** | 0.27 (2.08) | 0.23 (2.21) | 0.2950 (1.97) | 0.772 |
| **BBS ON** | 1 (2) | 0 (1) | 0.50(3) | 0.37 |
| **NFOG ON** | 0 (1) | 0 (2) | 0 (1) | 0.078 |
| **10- MWT Comfortable speed OFF** | 0.0021 (0.11) | 0.03 (0.12) | 0.0094 (0.08) | 0.704 |
| **10- MWT Comfortable speed ON** | 0.02 (0.16) | 0.04 (0.12) | 0.0062 (0.09) | 0.554 |
| **NMSS** | 0.50 (9) | -3.00 (14) | -3.50 (7) | 0.343 |
| 1-Cardiovascular | 0 (1) | 0 (2) | 0 (1) | 0.931 |
| 2-Sleep/fatigue | 0 (3) | 0 (4) | 0 (1) | 0.944 |
| 3-Mood/cognition | 0.50 (3) | -1 (2) | -1 (3) | 0.051 |
| 4-Perceptual problems/ Hallucinations | 0 (0) | 0 (0) | 0 (0) | 0.299 |
| 5-Attention/memory | -0.50 (2) | -1 (3) | 0 (3) | 0.711 |
| 6- Gastrointestinal tract | 0 (2) | -1 (2) | 0 (2) | 0.48 |
| 7-Urinary | 0 (3) | 0 (2) | 0 (1) | 0.528 |
| 8- Sexual function | 0 (1) | 0 (1) | 0 (0) | 0.091 |
| 9-Miscellaneous | 0 (3) | 0 (3) | -1 (2) | 0.329 |
| **BDI** | -1 (4) | 0 (4) | 0 (3) | 0.339 |
| **HAM-A** | 0.50 (2) | 0 (2) | 0 (2) | 0.898 |
| **PDQ -39 total** | 0.99 (6.63) | 3.22 (7.53) | 0.29 (4.04) | 0.495 |
| Mobility | 2.50 (7.50) | 2.50 (12.50) | 0 (7.50) | 0.771 |
| ADL | 2.08 (12.50) | 4.17 (14.58) | 4.17 (4.17) | 0.786 |
| Emotional wellbeing | 4.17 (14.58) | 0 (10.42) | 0 (4.17) | 0.234 |
| Stigma | 6.25 (12.50) | 6.25 (9.38) | 6.25 (10.94) | 0.534 |
| Social support | 0 (8.33) | 0 (16.67) | 0 (6.25) | 0.69 |
| Cognition | -3.13 (12.50) | 0 (12.50) | 0 (6.25) | 0.295 |
| Communication | -4.17 (14.58) | 0 (20.83) | 0 (8.33) | 0.365 |
| Bodily discomfort | 0 (25.00) | 0 (8.33) | 0 (16.67) | 0.833 |

Data are shown as median, (IQR).

**H&Y:**Hoehn & Yahr scale, **S&E-ADL:** Schwab and England-activities of daily living, **PIGD:**Postural instability and gait disorder, **10-MWT:** 10 meter walking test, **BBS:** Berg Balance Scale, **NFOG:**New freezing of gait questionnaire, **NMSS;** non motor symptoms scale, **BDI;** Beck Depression Inventory, **HAM-A;** Hamilton anxiety rating scale, **PDQ-39:** Parkinson disease questionnaire-39, **ADL:** activities of daily living, **ZNS:** Zonisamide

**Supplementary table 13**: Frequency of side effects among the three groups.

|  | **ZNS 25 mg**  **(no.=34)** | **ZNS 50 mg**  **(no.=26)** | **Placebo**  **(no.=35)** | **p value** | **ZNS 25 mg vs ZNS 50 mg** | **ZNS 25 mg vs placebo** | **ZNS 50 mg vs placebo** |
| --- | --- | --- | --- | --- | --- | --- | --- |
| **Total number of patients experienced side effects** | 17 (50%) | 17 (65.38%) | 8 (22.85%) | 0.003* | 0.233 | 0.019* | 0.001* |
| **Sedation** | 14 (41.17 %) | 13 (50 %) | 5 (14.28%) | 0.010* | 0.252 | 0.043* | 0.002* |
| **Anorexia** | 2 (5.88 %) | 3 (11.53 %) | 0 (0 %) | 0.134 |  | | |
| **Nausea** | 7 (20.58 %) | 5 (19.23%) | 1 (2.85 %) | 0.063 |  | | |
| **Epigastric pain** | 4 (11.76 %) | 2 (7.69 %) | 2 (5.71 %) | 0.656 |  | | |
| **Constipation** | 5 (14.7 %) | 3 (11.53 %) | 4 (11.42 %) | 0.902 |  | | |
| **Allergy, rash** | 0 (0 %) | 1 (3.84%) | 1 (2.85 %) | 0.546 |  | | |
| **Exacerbation of PD symptoms** | 1 (2.94 %) | 0 (0 %) | 0 (0 %) | 0.404 |  | | |
| **Hallucinations, psychosis** | 2 (5.88 %) | 0 (0 %) | 0 (0 %) | 0.160 |  | | |
| **Worsening or emergence of Dyskinesia** | 1 (2.94 %) | 3 (11.53 %) | 1 (2.85 %) | 0.243 |  | | |
| **Paresthesia** | 4 (11.76 %) | 3 (11.53 %) | 1 (2.85 %) | 0.329 |  | | |

Data are shown as number (%), **ZNS,** Zonisamide

*P value is* significant *if < 0.05*
